# Draft Genome Sequence of a Streptomycete Isolated from Potato Common Scab Lesions in the State of Sinaloa, Mexico

**DOI:** 10.1128/MRA.00827-18

**Published:** 2018-08-09

**Authors:** Amanda Alejo-Viderique, Luis Contreras-Castro, Rubén Félix-Gastélum, Luis A. Maldonado, Erika T. Quintana

**Affiliations:** a Laboratorio de Bioprospección en Actinobacterias, Escuela Nacional de Ciencias Biológicas (ENCB), Instituto Politécnico Nacional (IPN), Mexico City, Mexico; b Departamento de Ciencias Biológicas, Unidad Los Mochis, Universidad de Occidente, Los Mochis, Sinaloa, Mexico; c Facultad de Química, Universidad Nacional Autónoma de México (UNAM), Mexico City, Mexico; University of Maryland School of Medicine

## Abstract

Streptomyces sp. strain V2 was isolated from potato scab lesions in the state of Sinaloa, Mexico, and appears to be responsible for outbreaks in the area. The thaxtomin cluster was found in the ∼10.2-Mb genome; this cluster is associated with potato common scab disease in other potato pathogens.

## Abstract (precis)

Streptomyces sp. strain V2 was isolated from potato scab lesions in the state of Sinaloa, Mexico, and appears to be responsible for outbreaks in the area.

## ANNOUNCEMENT

The genus Streptomyces, class *Actinobacteria*, currently contains 533 described species, most of them isolated from soil (their primary natural habitat), although there are reports of species recovered from both freshwater and marine environments (1). Among this high number of described species, only a few are considered human and plant pathogens (2, 3).

Some of these plant pathogens cause economically important diseases, such as potato common scab (PCS), which appears as shallow or deep corky blemishes that disfigure the potato skin, which consequently needs excessive peeling (4). Streptomyces scabiei is regarded as the predominant PCS agent worldwide (5), although S. acidiscabies, S. turgidiscabies, S. europaeiscabiei, S. stelliscabiei, S. luridiscabiei, S. puniciscabiei, and S. niveiscabiei (6–9) have also been recovered from PCS lesions. These pathogenic strains have a polyphyletic nature and have been related by a transmissible pathogenicity island which seems to confer the pathogenic phenotype on some species. The main pathogenicity factor of this phenotype is the production of the phytotoxin thaxtomin, a nitrated dipeptide which inhibits cellulose synthesis in expanding plant tissue (10, 11).

Streptomyces sp. strain V2 was recovered as part of a study in the state of Sinaloa, Mexico, of the diversity of PCS lesions related to or associated with bacteria. At the time of writing, this ongoing study has recovered 22 actinobacterial strains identified by nearly complete 16S rRNA gene sequences and includes not only streptomycetes but also rare actinobacteria (i.e., Amycolatopsis and Lentzea spp.). Currently, studies are being conducted to establish either the pathogenic relationship of each isolate to the PCS lesion or its merely saprophytic role within the tubercle (A. Alejo-Viderique, E. Burgueño, L. A. Maldonado, G. Herrera, R. Felix, and E. T. Quintana, unpublished data).

The genome of strain V2 was sequenced by ChunLab (Seoul, South Korea) using the Illumina MiSeq sequencing platform. The obtained reads were assembled with SPAdes 3.1.1 (12). The genome size is 10.2 Mb. The GC content was found to be 71%. Two-way comparison of the average nucleotide identity (ANI) values (13) of S. scabiei and S. acidiscabies indicated values of 82.74% and 93.35%, respectively, suggesting that isolate V2 should constitute a novel species. The genome was annotated with the NCBI Prokaryotic Genome Annotation Pipeline (14). The number of genes was 9,222, with 68 tRNAs, 8 complete rRNAs, 3 noncoding RNAs, and 4 CRISPR arrays. Mining of the genome using antiSMASH 3.0 (15) found 53 potential secondary metabolite-related clusters. The antiSMASH suite predicted the presence of gene clusters related to the production of albaflavenone, alnumycin, ansamitocin, cahuitamycin, coelibactin, coelichelin, desferrioxamine B, desotamide, ectoine, furaquinocin A, gamma-butyrolactone, grincamycin, herboxidiene, hopene, informatipeptin, jawsamycin, kanamycin, kedarcidin, lactonamycin, laspartomycin, mensacaricin, nikkomycin, oxazolomycin, pactamycin, pristinamycin, salinamides, skyllamycin, and xantholipin, among others predicted by the Web tool NaPDos (16). It is worth mentioning that the phytotoxin thaxtomin cluster was also found, with over 50% of the genes showing similarity to the cluster of S. scabiei.

### Data availability.

This whole-genome shotgun project has been deposited in GenBank under the accession no. QFDR00000000 . The version described in this paper is the first version, QFDR01000000.
